# Supplementary material for: Lamotrigine Therapy for Bipolar Depression: Analysis of Self-Reported Patient Data
Source: JMIR Ment Health. 2018 Nov 22;5(4):e63. doi: 10.2196/mental.9026 (PMC6284142; doi:10.2196/mental.9026)
Supplement: Multimedia Appendix 1 [file mental_v5i4e63_app1.pdf]

Multimedia Appendix 1. Comparison of models: Logistic regression (LR), Linear Discriminant Analysis (LDA), Quadratic Discriminant Analysis (QDA), Linear Support Vector Machine (LSVM), Gaussian Kernel SVM(GSVM) and K-Nearest Neighbors(KNN). We show both the in-sample and out-of-sample classification accuracy.

FEATURE	AUC	IN SAMPLE ACCURACY						OUT OF SAMPLE ACCURACY					
		LR	LDA	QDA	LSVM	GSVM	KNN	LR	LDA	QDA	LSVM	GSVM	KNN
**QIDS-SR16 Mean(AVG)**	0.587	0.594	0.558	0.565	0.558	0.573	0.696	0.517	0.558	0.580	0.551	0.536	0.536
**QIDS-SR16 Standard Deviation(STD)**	0.552	0.558	0.544	0.551	0.544	0.558	1.000	0.406	0.536	0.500	0.551	0.500	0.478
**Low Frequency Power(LF)**	0.482	0.507	0.449	0.544	0.507	0.558	0.710	0.353	0.442	0.507	0.478	0.486	0.522
**High Frequency Power(HF)**	0.599	0.601	0.601	0.573	0.558	0.601	0.601	0.581	0.587	0.551	0.558	0.551	0.587
**DFA alpha**	0.587	0.573	0.544	0.565	0.544	0.565	0.645	0.418	0.536	0.544	0.544	0.464	0.529
**Coefficient of Variation(STD/AVG)**	0.614	0.609	0.587	0.587	0.580	0.594	0.630	0.604	0.587	0.594	0.580	0.573	0.580
**Power Ratio(HF/LF)**	0.571	0.544	0.507	0.507	0.515	0.573	0.616	0.555	0.471	0.478	0.471	0.536	0.580
**Skewness**	0.537	0.558	0.565	0.573	0.544	0.580	0.645	0.480	0.529	0.551	0.464	0.522	0.500
**Kurtosis**	0.581	0.616	0.558	0.558	0.551	0.587	0.652	0.580	0.551	0.551	0.565	0.587	0.587
**Slope of X_t vs X_{t-1}**	0.528	0.536	0.536	0.522	0.536	0.536	1.000	0.448	0.522	0.515	0.464	0.500	0.580
**Correlation coefficient of X_t vs X_{t-1}**	0.521	0.536	0.529	0.522	0.529	0.536	0.725	0.433	0.515	0.500	0.457	0.515	0.529
**Bipolar type**	0.511	0.507	0.507	0.507	0.507	0.507	0.507	0.360	0.442	0.457	0.442	0.471	0.493
**Age**	0.545	0.551	0.551	0.551	0.551	0.551	0.551	0.551	0.551	0.544	0.515	0.551	0.544
**Gender**	0.500	0.507	0.507	0.507	0.507	0.507	0.507	0.303	0.370	0.442	0.420	0.420	0.493
**All features combined**	0.720	0.696	0.674	0.732	0.659	1.000	0.580	0.557	0.536	0.493	0.558	0.449	0.536
**DFA alpha, Coeff. of Variation**	0.665	0.659	0.630	0.630	0.630	0.659	0.638	0.619	0.609	**0.623**	0.587	0.616	0.594
